# The Clinical Outcome Study for dysferlinopathy: An international multicenter study

**DOI:** 10.1212/NXG.0000000000000089

**Published:** 2016-08-04

**Authors:** Elizabeth Harris, Catherine L. Bladen, Anna Mayhew, Meredith James, Karen Bettinson, Ursula Moore, Fiona E. Smith, Laura Rufibach, Avital Cnaan, Diana X. Bharucha-Goebel, Andrew M. Blamire, Elena Bravver, Pierre G. Carlier, John W. Day, Jordi Díaz-Manera, Michelle Eagle, Ulrike Grieben, Matthew Harms, Kristi J. Jones, Hanns Lochmüller, Jerry R. Mendell, Madoka Mori-Yoshimura, Carmen Paradas, Elena Pegoraro, Alan Pestronk, Emmanuelle Salort-Campana, Olivia Schreiber-Katz, Claudio Semplicini, Simone Spuler, Tanya Stojkovic, Volker Straub, Shin'ich Takeda, Carolina Tesi Rocha, M.C. Walter, Kate Bushby, For the Jain COS Consortium

**Affiliations:** From The John Walton Muscular Dystrophy Research Centre (E.H., C.L.B., A.M., M.J., K. Bettinson, U.M., M.E., H.L., V.S., K. Bushby), Institute of Genetic Medicine, Newcastle upon Tyne, UK; Magnetic Resonance Centre (F.E.S., A.M.B.), Institute for Cellular Medicine, Newcastle University, UK; Jain Foundation, Inc. (L.R.), Seattle, WA; Division of Biostatistics and Study Methodology (A.C.), Center for Translational Science, Children's National Health System, Washington, DC; Department of Pediatrics, Epidemiology and Biostatistics (A.C.), George Washington University; Department of Neurology (D.X.B.-G.), Children's National Health System, Washington, DC; National Institutes of Health (NINDS) (D.X.B.-G.), Bethesda, MD; Carolinas Healthcare System Neurosciences Institute (E.B.), Charlotte; AIM & CEA NMR Laboratory (P.G.C.), Institute of Myology, Pitié-Salpêtrière University Hospital, Paris, France; Stanford University School of Medicine (J.W.D., C.T.R.), CA; Neuromuscular Disorders Unit (J.D.-M.), Department of Neurology, Hospital de la Santa Creu i Sant Pau, Barcelona, Spain; Centro de Investigación Biomédica en Red en Enfermedades Raras (CIBERER) (J.D.-M.), Barcelona, Spain; Muscle Research Unit (U.G., S.S.), Experimental and Clinical Research Center, A Joint Cooperation of the Charité Medical Faculty and the Max Delbrück Center for Molecular Medicine, Berlin, Germany; Washington University (M.H., A.P.), St. Louis, MO; Institute for Neuroscience and Muscle Research (K.J.J.), Children's Hospital at Westmead, University of Sydney, Australia; Nationwide Children's Hospital (J.R.M.), Columbus, OH; Department of Neurology (M.M.-Y., S.T.), National Center Hospital, National Center of Neurology and Psychiatry, Tokyo, Japan; Neuromuscular Unit, Department of Neurology (C.P.), Hospital U. Virgen del Rocío, Instituto de Biomedicina de Sevilla, Spain; Department of Neuroscience (E.P., C.S.), University of Padova, Italy; Neuromuscular and ALS Center (E.S.-C.), La Timone Hospital, Aix-Marseille Université, France; Department of Neurology (O.S.-K., M.C.W.), Friedrich-Baur-Institute, Ludwig-Maximilians-University of Munich, Germany; and Institut de Myologie (T.S.), AP-HP, G.H. Pitié-Salpêtrière, Boulevard de l'Hôpital, Paris, France.

## Abstract

**Objective:**

To describe the baseline clinical and functional characteristics of an international cohort of 193 patients with dysferlinopathy.

**Methods:**

The Clinical Outcome Study for dysferlinopathy (COS) is an international multicenter study of this disease, evaluating patients with genetically confirmed dysferlinopathy over 3 years. We present a cross-sectional analysis of 193 patients derived from their baseline clinical and functional assessments.

**Results:**

There is a high degree of variability in disease onset, pattern of weakness, and rate of progression. No factor, such as mutation class, protein expression, or age at onset, accounted for this variability. Among patients with clinical diagnoses of Miyoshi myopathy or limb-girdle muscular dystrophy, clinical presentation and examination was not strikingly different. Respiratory impairment and cardiac dysfunction were observed in a minority of patients. A substantial delay in diagnosis was previously common but has been steadily reducing, suggesting increasing awareness of dysferlinopathies.

**Conclusions:**

These findings highlight crucial issues to be addressed for both optimizing clinical care and planning therapeutic trials in dysferlinopathy. This ongoing longitudinal study will provide an opportunity to further understand patterns and variability in disease progression and form the basis for trial design.

Dysferlinopathy is a term for a group of rare muscular dystrophies with recessive mutations in the *DYSF* gene, which encodes the skeletal muscle protein dysferlin.^1,2^ Two major phenotypes are Miyoshi myopathy (MM),^3^ presenting with distal weakness and limb-girdle muscular dystrophy type 2B (LGMD2B),^4,5^ affecting more proximal muscles. Other reported phenotypes include the more rapidly progressive distal myopathy with anterior tibial involvement,^6^ proximodistal weakness, and a pseudometabolic presentation.^7,8^

Several studies have reviewed the phenotypes of dysferlinopathy demonstrating a high degree of variability in the initial pattern of weakness. Symptom onset in young adulthood, highly elevated serum creatine kinase (CK), and characteristic MRI pattern are generally consistent.^2,8–14^ However, patients with atypical features are reported and the full spectrum of dysferlinopathy phenotypes and patterns of disease progression is yet to be fully described.^15–17^

As the neuromuscular field moves toward trial readiness, a clearer understanding of the natural history of these rare diseases is essential. This report describes baseline characteristics of participants in the Jain Foundation–funded clinical outcome study—a large cohort of patients with dysferlinopathy, enabling characterization of common and rarer phenotypic features. This work will form the baseline for longitudinal assessment aiming to define distinct disease trajectories and a robust set of outcome measures for clinical trials and to identify areas for improving clinical practice.

## METHODS

Inclusion criteria were ≥2 pathogenic mutations in *DYSF*, or 1 pathogenic mutation plus either absent dysferlin expression on immunoblot (IB)^18^ or ≤20% dysferlin monocyte expression (ME).^19^ Truncating mutations and splice-site mutations affecting the +1/−1 or 2 positions were deemed pathogenic. Pathogenicity of other splice-site mutations and missense mutations were defined according to the UMD Predictor (http://umd-predictor.eu).

Patients have 6 visits over 3 years (screening, baseline, 6 months, 1, 2, and 3 years). At each visit, a medical examination is conducted, and quality of life, exercise, and medical history data are collected via questionnaires. Blood is drawn for hematologic and biochemical assays. Patients can choose to provide DNA, RNA, serum, plasma, and skin biopsy for biobanking. Cardiac assessment by ECG and echocardiogram are performed at baseline and 3 years. MRI assessment (to be reported separately) includes lower limb T1W, T2, 3-point Dixon (lower limb), and magnetic resonance spectroscopy evaluation (3 sites) at baseline, 1, 2, and 3 years.

Physiotherapists, trained and assessed in investigator meetings, perform evaluations at each visit. They assess respiratory function (sitting forced vital capacity [FVC]), muscle strength (Manual Muscle Testing [MMT]), and functional status (adapted North Star Ambulatory Assessment [a-NSAA] in ambulant patients, timed tests [rise from floor (RFF), 10-m walk/run, 4 stair climb and descend, Timed Up and Go (TUG)], and 6-minute walk). Assessments were reviewed for consistency between screening and baseline by lead physiotherapists from Newcastle.

The a-NSAA is based on the validated NSAA, a 17-item scale with a maximum score of 34 used in Duchenne muscular dystrophy. This was adapted adding items relevant to ambulatory ability in dysferlinopathy creating a 22-item scale with a maximum score of 51 (table e-1 at Neurology.org/ng).

Using a-NSAA and ambulatory status, the cohort was stratified into mild (a-NSAA 40–51), moderate (a-NSAA 6–39), or severe (a-NSAA ≤5 or nonambulant) groups. Ambulation status was determined by the ability of patients to walk 10 m with shoes and usual walking aids or orthotics. Medical Research Council (MRC) power grades, timed tests, and respiratory status were reported according to this stratification.

For analysis, 5-point MRC power grades for MMT were converted to an 11-point scale (0, 1, 2, 3−, 3, 3+, 4−, 4, 4+, 5−, and 5).

Statistical analysis was performed using Prism software (GraphPad Software Ltd., La Jolla, CA). Demographics were collected for ethnicity, sex, age, ambulatory status, years symptomatic, and mutation details. Median values and ranges were calculated for the number of years symptomatic, age at symptom onset, age at diagnosis, and MMT analysis. Mean values (SD and ranges) were calculated for serum CK, serum creatinine, and serum urea. Percent predicted FVC and timed tests (10-m run, TUG, RFF, stair ascend, descend, and 6-minute walk test) are stratified by disease severity. MMT median values were also stratified by disease severity and analyzed for symmetry between right and left, anterior and posterior, and upper and lower limb muscle groups using the Wilcoxon signed-rank test, considering a *p* value of less than 0.05 statistically significant.

### Standard protocol approvals, registrations, and patient consents.

All study participants provided informed consent. The study was approved by ethical review boards in each country.

## RESULTS

### Study demographics.

Included were 193 patients from 15 sites (Newcastle, Barcelona, Seville, Munich, Berlin, Padova, Marseille, Paris, Saint Louis, Columbus, Charlotte, Washington, DC, Stanford, Tokyo, and Sydney) representing 8 countries (United Kingdom, Spain, Germany, Italy, France, the United States, Japan, and Australia). Participants' ethnicities were white (71%), Asian (17%), black (3%), Hispanic (6%), and other (3%). Participants were 52% female and 48% male. Ages range from 12 to 88 years (mean age 40 years). Participants were 75% ambulant (36% male/39% female) and 25% nonambulant (13% male/12% female). At assessment, the majority reported symptoms for 25 years or less (77%). Median symptom duration was 17 years (range 3–52 years).

### Genetic and protein expression findings.

In total, 175 different mutations were observed (table e-2), 112 only in a single individual; 49.2% of mutations were truncating: 32.8% frameshift and 16.4% nonsense. The remainder were missense (32.8%), splice-site (17.5%), or in-frame duplication (0.6%).

Mutations were widely distributed throughout the gene. Table 1 shows the most frequently observed mutations. Two previously reported founder mutations were identified: c.2779delG,^20^ in 3 individuals with Hispanic ethnicity and c.5713C>T,^21^ observed in 4 individuals of diverse ethnicities.

**Table 1.**
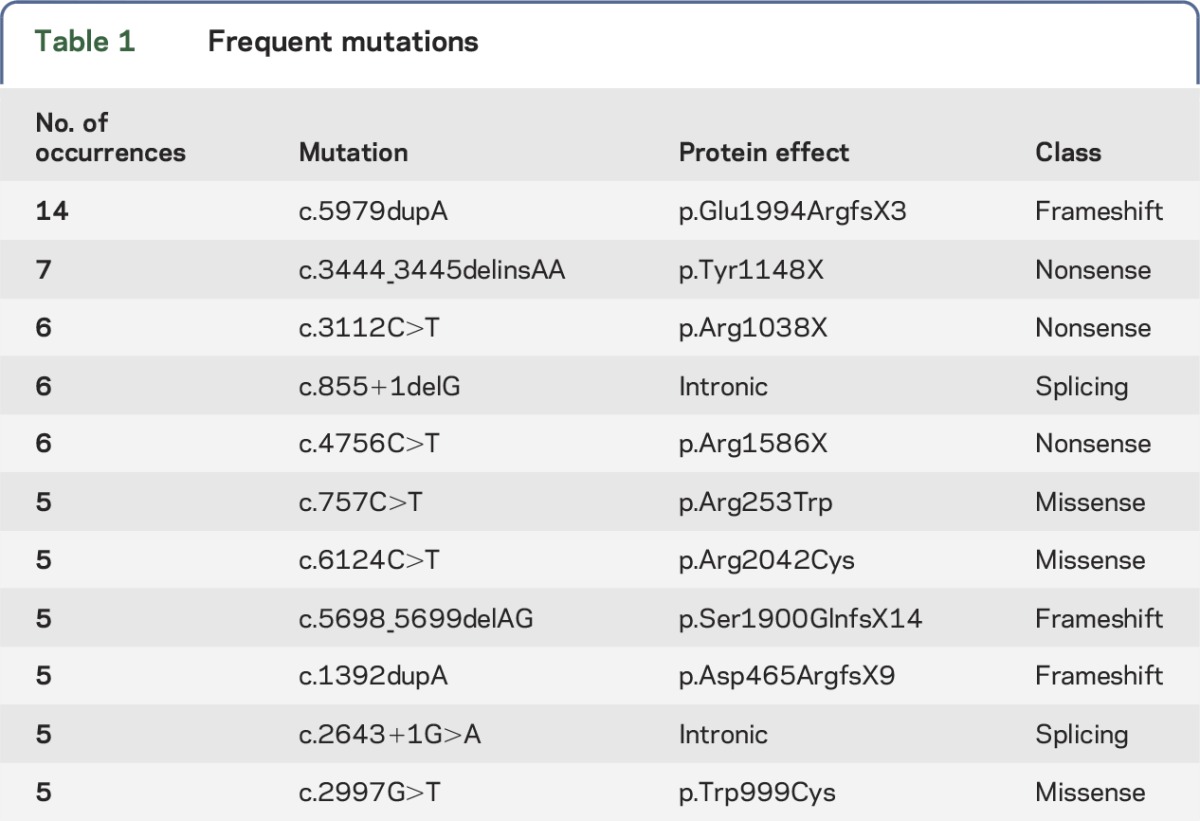
Frequent mutations

Eighty-four percent of participants had 2 pathogenic mutations in *DYSF*. Thirteen percent had a single heterozygous mutation and absent or reduced (i.e., <20%) dysferlin expression on IB or ME. Three percent had >2 pathogenic mutations.

Dysferlin ME, IB, or immunohistochemistry (IH) data were available for 153 (79%) patients. Of these, 68% had absent and 30% had reduced dysferlin expression. Symptom onset age did not vary according to dysferlin expression levels. Normal dysferlin expression was observed in 3 individuals who presented with moderate or severe disease.

Of the 40 patients (21%) with no protein expression data, 28 had 2 clearly deleterious mutations (frameshift, splice-site, or nonsense), 8 had 1 clearly deleterious mutation and 1 missense mutation that was predicted to be pathogenic by the UMD predictor, and 4 patients had 2 missense mutations predicted to be pathogenic. There was no relationship between genotype (homozygosity for missense, splicing, or truncating mutations) and protein expression, age at onset, or disease severity.

### Symptom onset and diagnosis.

**Figure 1.**
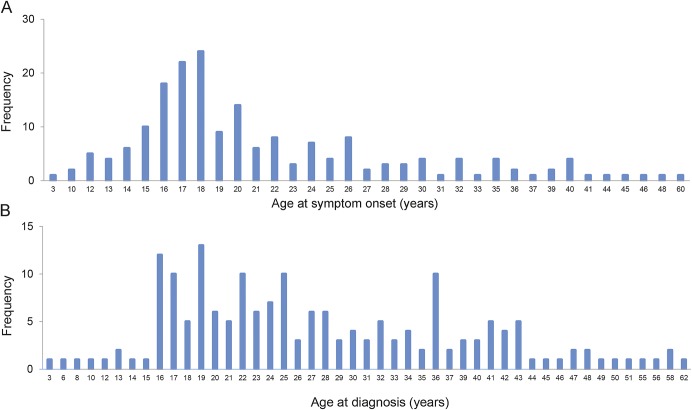
Age of patients at symptom onset and diagnosis The mean time from onset to diagnosis was 6 years.

Self-reported age at “first muscle symptoms” ranged from 3 to 60 years (median 19 years) (figure 1). Most patients had symptoms preceding diagnosis; however, 24% were diagnosed after an incidental finding of elevated CK and 13% after diagnosis in a relative.

Initial symptoms varied, with some patients reporting multiple symptoms. Most commonly reported was lower limb weakness (72%); this was proximal (15%), distal (32%), or both (25%). Upper limb weakness was less common (7%). Others described muscle wasting (27%—predominantly distal lower limbs), pain, stiffness, or cramps (13%), or pseudohypertrophy (6%—predominantly distal lower limbs). Seventeen percent described onset following trauma or illness, but the majority described symptom onset over months.

Prior to symptom onset, 80% reported frequent participation in sports; daily (13%) or several times a week (42%). Forty-four percent reported “average” sporting ability, with 19% competing at the regional or national level.

The median age at confirmed diagnosis was 25 years (range 3–62 years) (figure 1). Mean time from symptom onset to diagnosis in the 1970s was 20.5 years (SD 10.7), falling to 3.1 years (SD 2.6) with onset in 2000s. Patient-reported clinical diagnoses were LGMD2B (60%), MM (30%), proximodistal dysferlinopathy (6%), hyperCKemia (3%), and “other” including paravertebral muscular dystrophy or pseudometabolic dysferlinopathy (2%). Clinical diagnosis varied by research site but not by patient ethnicity or age at symptom onset. MM was the most common diagnosis in Japan: odds ratio (OR) 7.01 (2.10–23.46) and LGMD2B in England: OR 6.12 (2.28–16.25).

An initial diagnosis of polymyositis was reported by 16%, and 25% reported previous corticosteroid use. There were geographical differences in prior steroid use, with none in Australia and >60% in Germany, possibly because of a previous clinical trial.^22^

**Figure 2.**
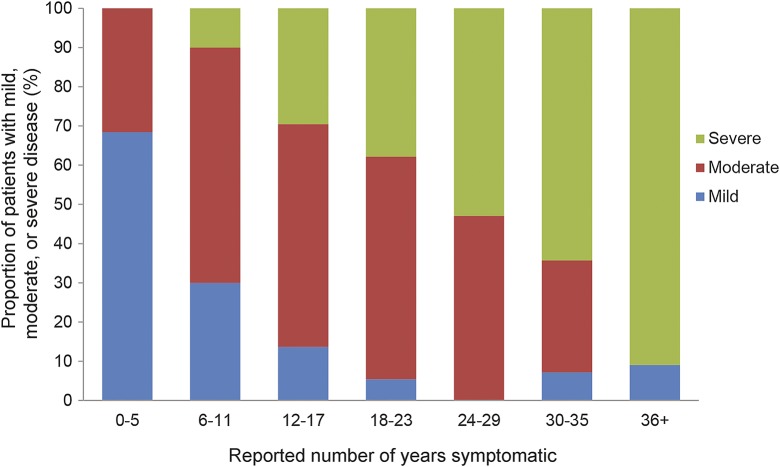
Patient stratification by the reported duration of symptoms and disease severity at the time of assessment The percentage of patients within each severity category is given. Severity is defined as mild if the adapted North Star Ambulatory Assessment score is 40–51, moderate: 6–39, severe: 5 or less or nonambulatory. Symptomatic patients for whom sufficient data were available to assign severity were included (n = 182). Numbers of patients within each category are as follows: mild n = 34, moderate n = 89, severe n = 59.

The longer the duration of symptoms, the greater proportion of patients with severe disease (figure 2). However, 2 patients with symptoms for over 30 years remain mildly affected. Four patients, aged 16 to 30, reported no muscle symptoms.

### Physical examination.

Thirty-six percent of patients had joint contractures, commonly affecting ankles, knees, and elbows. Muscle wasting was observed in 80% of patients most commonly in distal lower limbs (71%). Pseudohypertrophy was noted in 11%; usually in distal lower limbs but sometimes proximal lower limbs, upper arms, shoulders, or neck. Additional features observed include scoliosis (8%), rigid spine (7%), tremor (5%), facial weakness (3%), tongue fasciculations (3%), dysarthria (0.5%), or myotonia (0.5%).

### Clinical investigations.

Mean serum CK at baseline was 4,562 IU/L (SD 3,937; range 209–23,124 IU/L) with values falling with increasing age and disease duration. Serum creatinine was abnormally low in 70% of patients (mean 36.7 μmol/L; range 11–145 μmol/L, SD 18), likely reflecting reduced muscle mass. Mean serum urea was within normal range (mean 6.2 mmol/L; range 1.1–23.9 mmol/L and SD 3.4 mmol/L). Elevated alanine aminotransferase (91%) and aspartate transaminase (93%) levels were seen in most patients, consistent with muscular dystrophy. Elevated alkaline phosphatase (6%) and total bilirubin (9%) was less common.

### Baseline MMT.

**Figure 3.**
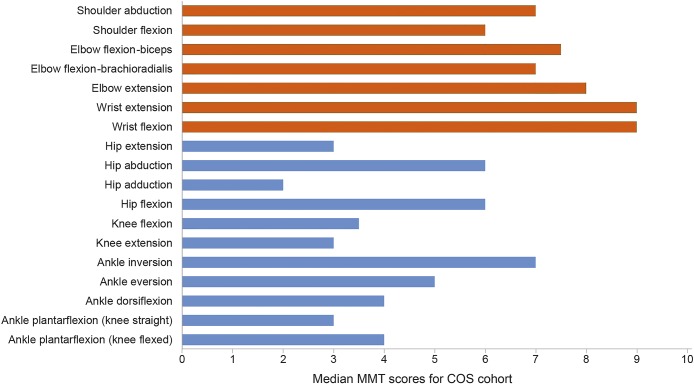
Comparison of median manual muscle test scores in the upper and lower limbs Data were available for 189 study participants. The 5-point Medical Research Council power grade was converted to an 11-point scale (0, 1, 2, 3−, 3, 3+, 4−, 4, 4+, 5−, and 5). Observed Manual Muscle Testing scores ranged from 0 or 1 to 10 for each movement assessed, with the exception of wrist extension for which the lowest observed score was 2. Overall, the most severely affected muscle groups were hip adduction, extension, knee flexion and extension, and ankle plantar flexion, dorsiflexion, and eversion. The least severely affected muscle groups were wrist flexion and extension. Red indicates the upper limb muscles and blue indicates the lower limb muscles. COS = Clinical Outcome Study.

The characteristics of the median MMT values are summarized in figure 3. Strength was better preserved in upper limbs than lower limbs: median MMT scores 8/10 vs 3/10, respectively (*p* ≤ 0.0001) There was no asymmetry between right and left in any muscle group. Analysis of MMT scores in posterior vs anterior muscle groups in the lower limbs demonstrated a difference in hip muscles with hip flexion stronger than extension (hip flexion score 6/10, extension 3/10 [*p* < 0.0001, Wilcoxon signed-rank test]).

MMT was evaluated depending on disease severity scores. In the mild cohort, median MRC power was ≥4 in all muscle groups, with lower limbs generally weaker than upper limbs. Elbow and wrist flexion and extension, knee extension, and ankle inversion, eversion, and dorsiflexion were typically of normal power. In the moderate cohort, on average, lower limbs were weaker in all muscle groups than upper limbs with median MRC scores of ≤4 and ≥4, respectively. Hip adduction was weakest (median MRC 2.5), and wrist flexion and extension were strongest (median MRC 5−). In the severe cohort, lower limb proximal and distal muscle groups were similarly affected (median MRC 1 or 2, with the exception of hip abduction MRC 3−). Ankle dorsiflexion, eversion, and plantar flexion were weakest (median MRC grade 1). In upper limbs, there was proximal weakness (median MRC 3−) with distal strength preservation (wrist flexion/extension median MRC 4−/4+).

When baseline timed tests were stratified by disease severity (table 2), there was overlap between groups, indicating the variability of physical ability within the cohort.

**Table 2.**
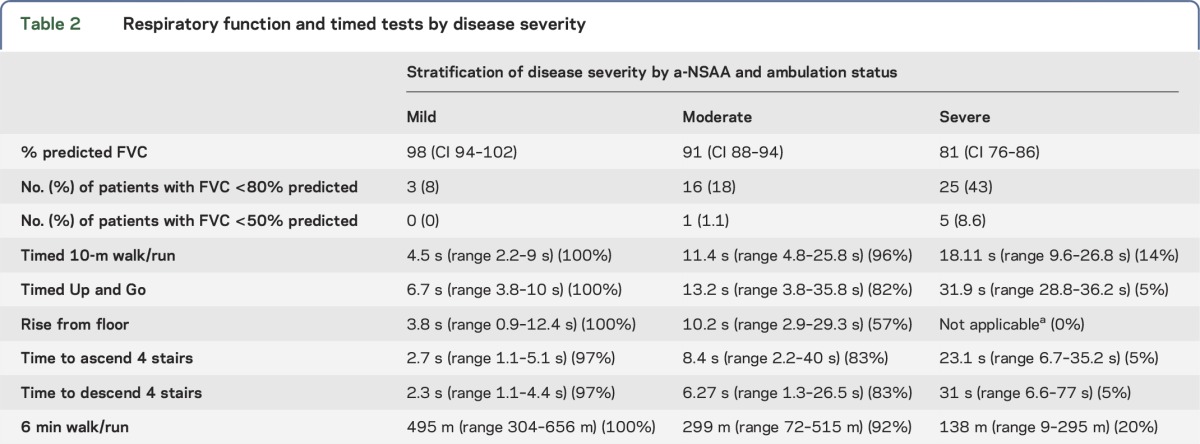
Respiratory function and timed tests by disease severity

### Cardiac findings.

Impaired left ventricular function, defined as ejection fraction <55%, was detected by study-related echocardiography in 7 patients (aged 29–69) and will be further evaluated by cardiac MRI. To date, 3 of these are completed—2 are normal and 1 confirms cardiomyopathy (patient aged 51). One additional patient had cardiomyopathy diagnosed prior to the study, at age 46 years. Of the 2 patients with cardiomyopathy, one had reduced FVC at 67% predicted and used nocturnal ventilation.

### Respiratory findings.

Increased disease severity was associated with lower FVC (table 2). Nocturnal noninvasive ventilation was used by 4 patients, all of whom reported a diagnosis of sleep apnea, and 3 of whom had a body mass index of >30. Disease severity in these 4 individuals ranged from mild to severe disease with predicted FVC between 50% and 82%.

### Previous diagnosis.

Clinical features were evaluated by preexisting clinical diagnosis. All patients with a clinical diagnosis of hyperCKemia fell into the mild category. Patients diagnosed as LGMD2B or MM were seen in mild, moderate, and severe groups. Median MMT values were similar between LGMD2B and MM groups, with proximal and distal lower limb weakness and predominantly proximal upper limb weakness. Ankle inversion was better preserved in LGMD2B patients (median MRC 4− vs median MRC 2 in MM). Patients diagnosed with proximodistal dysferlinopathy were more severely affected, with weakness extending to distal upper limbs (wrist extension median MRC 2, wrist flexion median MRC 3), and none had mild disease. Mean symptom duration was 17 years for the whole cohort. Apart from hyperCKemia, with median 5 years, this did not differ according to clinical diagnosis.

## DISCUSSION

We report the initial findings of an international observational study of patients with genetically confirmed dysferlinopathy. This cross-sectional analysis of a large and geographically diverse cohort of patients highlights both typical features and disease course, and outlying characteristics. This will form the basis for future longitudinal analysis of clinical outcomes, cardiac and respiratory evaluations, and muscle MRI data (the latter being reported separately).

Inclusion criteria for this study aimed to replicate the strict diagnostic criteria required for clinical trials: all patients have 2 mutations or a heterozygous mutation with additional evidence of absent or disease-range dysferlin protein expression by ME or IB.

Genetic data from this cohort support the high degree of genetic heterogeneity reported previously.^2,7,23^ One-third of patients have nonsense mutations, indicating that nonsense read-through therapies, now licensed in Europe for use in Duchenne muscular dystrophy, may be a potential therapy for some patients with dysferlinopathy.

A high percentage of the mutations were missense mutations. Although usually associated with absent or reduced dysferlin expression (table e-2), further analysis is needed to determine the mechanism by which these missense mutations lead to disease. Some investigations link missense mutations to protein instability causing reduced dysferlin levels.^24^ Others have demonstrated that missense mutations can lead to normal protein expression levels, but abnormal protein localization, which results in clinical disease.^25^ Assays for the functionality of dysferlin protein with various missense mutations are currently being investigated by the Jain Foundation and may help to refine the diagnostic process in the future.

Most patients included in this study have absent or reduced dysferlin on IH, IB, or ME. Absence of dysferlin was more commonly noted than reduction, irrespective of the severity of the clinical phenotype or mutation type. We identified 3 patients with 2 *DYSF* missense mutations in whom dysferlin protein levels were normal. The typical diagnostic procedure for dysferlinopathy diagnosis has been to identify absent or reduced dysferlin protein levels and then sequence the dysferlin gene. Therefore, patients with pathogenic *DYSF* mutations and normal dysferlin protein levels are rarely identified.^26^ As genetic testing becomes more prevalent as a first-line investigation, patients with normal dysferlin levels may be increasingly recognized and caution will be required before generalizing results from this study to that population.

More than 2 dysferlin mutations were found in 3% of cases. However, aside from one novel mutation (c.6056G>A), all of these missense mutations have previously been associated with reduced dysferlin expression when in the homozygous state or in combination with one other mutation,^7,25–29^ which supports their pathogenicity.

We identified that time from symptom onset to diagnosis has reduced. As our data indicate that 30% of patients are moderately affected within 5 years, earlier diagnosis is likely to reduce unnecessary testing or potentially detrimental steroid treatment.^22^ As therapies become available, any delay becomes more costly because the window of opportunity to treat may be missed. We hope improved awareness and delineation of the dysferlinopathy phenotype will continue to improve time to diagnosis.

Dysferlinopathy is often assigned a particular clinical phenotype, most commonly MM or LGMD. The pattern of weakness between patients given these 2 diagnoses did not differ in our study. A clinical diagnosis of proximodistal dysferlinopathy was associated with more severe disease, and this appears unrelated to symptom duration. The 3% patients labeled as hyperCKemia had symptoms for a shorter duration. Longitudinal study will clarify whether this is a presentation of early disease or a distinct phenotype. We noted a number of occasionally reported features, such as tremor or dysarthria, the significance of which is unclear. Above-average sporting ability before symptom onset has been reported previously in dysferlinopathy^10^ and is supported here with 19% of our cohort participating in sport at the regional or national level. The basis for this remains unknown.

We stratified patients by a-NSAA score and ambulation status into mild, moderate, and severely affected. This baseline analysis has demonstrated that weakness predominantly affects lower limbs in both proximal and distal muscle groups, regardless of disease severity. Increasing proximal upper limb weakness is connected with more severe disease. An increasing proportion of patients are more severely affected with increasing symptom duration. The rate of disease progression is variable, with >30% of patients mildly or moderately affected ≥30 years from symptom onset, while a similar proportion are severely affected after ≤17 years of symptoms (figure 2). The cause of this variability is not known, but differing presentations within a single family or common genotypes suggest the presence of disease-modifying factors.^30,31^ Given this highly variable severity, pattern of weakness and rate of progression in dysferlinopathy, we anticipate that longitudinal data will help to elucidate potentially distinct disease trajectories.

We observed that 6 patients with moderate or severe disease had an FVC <50%, supporting the need for respiratory function monitoring in moderate or severe disease.^32^ Four patients used nocturnal ventilation and reported sleep apnea. This may be coincidental as all have FVC ≥50% and 3 patients have body mass index >30. Two patients were identified with cardiomyopathy. Echocardiogram analysis for left ventricular dysfunction will be explored further in this study. Cardiac abnormalities have previously been reported in dysferlinopathy, but whether these are a consequence of dysferlinopathy or an alternative etiology is not established.^9,32–35^ Low serum creatinine seen in 70% of patients is relevant for renal function monitoring, as creatinine-dependent methods will be uninformative.^36^

This analysis has identified a number of findings pertinent to the clinical care and planning of trials for patients with dysferlinopathy. Diagnosis is frequently delayed. Detailed analysis of muscle strength and function across different clinical diagnoses suggests that distinctions in pattern of weakness between MM, LGMD2B, and other phenotypes are limited. Emerging longitudinal data will allow us to assess whether progression of weakness is also similar, allowing patients with different clinical diagnoses to be considered as a whole in planning clinical studies. A small proportion of patients have respiratory dysfunction and cardiac abnormalities. Although the general phenotype is of a slowly progressive disease manifesting in young adulthood, there are patients with disease onset at extremes of age and divergent rates of progression. The etiology of this variability is unclear but important to understand for clinical trials and developing validated outcome measures. As longitudinal data on this cohort emerge, we anticipate being able to contribute to the trial readiness of this patient group.

## Supplementary Material

Data Supplement

Coinvestigators

## GLOSSARY

a-NSAA: adapted North Star Ambulatory Assessment
CK: creatine kinase
FVC: forced vital capacity
IB: immunoblot
IH: immunohistochemistry
LGMD: limb-girdle muscular dystrophy
LGMD2B: limb-girdle muscular dystrophy type 2B
ME: monocyte expression
MM: Miyoshi myopathy
MMT: Manual Muscle Testing
MRC: Medical Research Council
NSAA: North Star Ambulatory Assessment
OR: odds ratio
RFF: rise from floor
TUG: Timed Up and Go
