# Supplementary material for: The Clinical Outcome Study for dysferlinopathy: An international multicenter study
Source: Neurol Genet. 2016 Aug 4;2(4):e89. doi: 10.1212/NXG.0000000000000089 (PMC4994875; doi:10.1212/NXG.0000000000000089)
Supplement: Data Supplement [file supp_2_4_e89__index.html]

Data Supplement 

# The Clinical Outcome Study for dysferlinopathy

## Data Supplement

**Files in this Data Supplement:**

- Table e-1 - Microsoft Word file
- Table e-2 - Microsoft Word file
