# Supplementary material for: The Clinical Outcome Study for dysferlinopathy: An international multicenter study
Source: Neurol Genet. 2016 Aug 4;2(4):e89. doi: 10.1212/NXG.0000000000000089 (PMC4994875; doi:10.1212/NXG.0000000000000089)
Supplement: Data Supplement [file supp_2.4.e89_table_e-1.docx]

**Supplementary table 1 – participant mutations and protein expression data**

This table lists all patients in the COS study. The mutation and protein change information is based on the reference sequence NM_ 003494. Protein expression information was collected from the time of diagnosis. It was a requirement for patients with only 1 mutation but not all patients with 2 or more mutations had expression information available. Pathogenicity of missense mutations was ascertained by the UMD predictor – a score of <65 is ‘probably a polymorphism’, 65-74 ‘probably pathogenic’ and >75 ‘pathogenic’. To support the pathogenicity of missense mutations, the background frequency of these mutations were also checked in the broad institutes genome browser (**exac**.broadinstitute.org). The table lists the number of people in the broad institutes sample population of 60,706 unrelated individuals who are homozygous for this mutation followed by the frequency of finding this mutation in a single allele in this population.

| **ID** | **no. of mutations**  **heterzygous/**  **homozygous** | **Mutation nomenclature** | **Associated protein change (if available)** | **Type of mutation** | **Muscle biopsy - immunohistochemistry** | **Muscle biopsy - western blot** | **monocyte expression** | **EXAC frequency (no homozygotes, allele frequency)** | | **UMD score** |
| --- | --- | --- | --- | --- | --- | --- | --- | --- | --- | --- |
| **1** | 1-het | c.353delT | p.Val118AlafsX33 | frameshift | absent | absent | NP |  |  | |
| **2** | 1-het | c.3517dupT | p.Ser1173PhefsX2 | frameshift | NP | absent | NP |  |  | |
| **3** | 1-het | c.3112C>T | p. Arg1038X | nonsense | NP | NP | absent |  |  | |
| **4** | 1-het | c.2779delG | p.Ala927LeufsX21 | frameshift | absent | NP | absent |  |  | |
| **5** | 1-het | c.5903G>A | p.Trp1968X | nonsense | absent | NP | absent |  |  | |
| **6** | 1-het | c.2875C>T | p.Arg959Trp | missense | NP | NP | absent | 0, 0.0001 | 94 | |
| **7** | 1-het | c. 2163-2A>G |  | splice site | NP | NP | absent |  |  | |
| **8** | 1-het | c.4360G>T | p.Gln1454X | nonsense | absent | absent | absent |  |  | |
| **9** | 1-het | c.5159delG | p.Arg1720LeufsX2 | frameshift | NP | NP | 8.90% |  |  | |
| **10** | 1-het | c.5159delG | p.Arg1720LeufsX2 | frameshift | NP | NP | 7.50% |  |  | |
| **11** | 1-het | c.863dupA | p.Asp288GlufsX40 | frameshift | absent | absent | NP |  |  | |
| **12** | 1-het | c.2997G>T | p.Trp999Cys | missense | absent | absent | NP | 0, 0.00002 | 71 | |
| **13** | 1-het | c.5871_5872delGT | p.Ser1958ProfsX3 | frameshift | reduced | absent | NP |  |  | |
| **14** | 1-het | c.2810+1G>A |  | splice site | absent | absent | NP |  |  | |
| **15** | 1-het | c.3516-3517delTT | p.Ser1173X | nonsense | absent | absent | NP |  |  | |
| **16** | 1-het | c.89_236del | p.Val31SerfsX71 | frameshift | NP | NP | absent |  |  | |
| **17** | 1-het | c.2643+5G>A | splice site | splice site | NP | NP | absent |  |  | |
| **18** | 1-het | c.3517dupT | p.Ser1173PhefsX2 | frameshift | absent | absent | NP |  |  | |
| **19** | 1-het | c.4090C>T | p.Gln1364X | nonsense | NP | NP | absent |  |  | |
| **20** | 1-het | c.3035G>A | p.Trp1012X | nonsense | absent | absent | NP |  |  | |
| **21** | 1-het | c.5526-2A>G |  | splice site | normal | absent | NP |  |  | |
| **22** | 1-het | c.5526-2A>G |  | splice site | normal | absent | NP |  |  | |
| **23** | 1-het | c.5698_5699delAG | p.Ser1900GlnfsX14 | frameshift | NP | NP | absent |  |  | |
| **24** | 1-het | c.3065G>A | p.Arg1022Gln | missense | NP | absent | NP | 29, 0.02 | 65 | |
| **25** | 1-het | c.742C>T | p.Gln248X | nonsense | NP | NP | absent |  |  | |
| **26** | 1-het | c.1639-6T>A |  | splice site | absent | NP | absent |  |  | |
| **27** | 2-hom | c.757C>T | p.Arg253Trp | missense | reduced | absent | NP | 0, 0.0001 | 93 | |
| **28** | 2-hom | c.1167_1180dup | p.Met394ArgfsX10 | frameshift | reduced | reduced | NP |  |  | |
| **29** | 2-hom | c.1642delG | p.Glu548LysfsX79 | frameshift | reduced | absent | NP |  |  | |
| **30** | 2-hom | c.2163-1G>T |  | splice site | normal | absent | NP |  |  | |
| **31** | 2-hom | c.175delC | Leu59TrpfsX92 | frameshift | reduced | absent | NP |  |  | |
| **32** | 2-hom | c.2163-1G>T |  | splice site | reduced | absent | NP |  |  | |
| **33** | 2-hom | c. 4882G>A | p.Gly1628Arg | missense | absent | absent | absent | 0,0 | 88 | |
| **34** | 2-hom | c.4354C>A | p.Pro1452Thr | missense | absent | absent | absent | 0,0 | 80 | |
| **35** | 2-hom | c.2875C>T | p.Arg959Trp | missense | absent | reduced | NP | 0, 0.0001 | 94 | |
| **36** | 2-hom | c.4934T>A | p.Ile1645Asn | missense | absent | NP | NP | not listed | 100 | |
| **37** | 2-hom | c. 2779delG | p.Ala927LeufsX21 | frameshift | absent | reduced | 0.20% |  |  | |
| **38** | 2-hom | c.1555G-C | p.Gly519Arg | missense | absent | NP | absent | 0,0 | 100 | |
| **39** | 2-hom | c.1555G-C | p.Gly519Arg | missense | absent | NP | absent | 0,0 | 100 | |
| **40** | 2-hom | c.5979dupA | p.Glu1994ArgfsX3 | frameshift | NP | NP | NP |  |  | |
| **41** | 2-hom | c.5979dupA | p.Glu1994ArgfsX3 | frameshift | absent | NP | NP |  |  | |
| **42** | 2-hom | c.3904_4410del | p.Ser1302_Gln1470del | inframe deletion | absent | absent | NP |  |  | |
| **43** | 2-hom | c.1852G>A | p.Gly618Arg | missense | absent | absent | NP | 0, 0.00002 | 94 | |
| **44** | 2-hom | c.663+1G>C |  | splice site | NP | NP | NP |  |  | |
| **45** | 2-hom | c.2551C>T | p.Gln851X | nonsense | NP | NP | NP |  |  | |
| **46** | 2-hom | c.4497delT | p.Phe1499LeufsX4 | frameshift | NP | NP | NP |  |  | |
| **47** | 2-hom | c.4756C>T | p.Arg1568X | nonsense | absent | absent | NP |  |  | |
| **48** | 2-hom | c.3112C>T | p.Arg1038X | nonsense | reduced | absent | NP |  |  | |
| **49** | 2-hom | c.895G>T | p.Gly299Trp | missense | NP | absent | NP | not listed | 94 | |
| **50** | 2-hom | c.4022T>C | p.Leu1341Pro | missense | NP | absent | NP | 0, 0.00002 | 71 | |
| **51** | 2-hom | c.1168_1180+1dup14 |  | frameshift | absent | NP | absent |  |  | |
| **52** | 2-hom | c.89_236del | p.Val31SerfsX71 | frameshift | NP | NP | NP |  |  | |
| **53** | 2-hom | c.1368C>A | p.Cys456X | nonsense | absent | absent | NP |  |  | |
| **54** | 2-hom | c.5429G>A | p.Arg1810Lys | missense | normal | NP | NP | not listed | **76** | |
| **55** | 2-hom | c.663+1G>A |  | splice site | absent | absent | NP |  |  | |
| **56** | 2-hom | c.5341-1G>A |  | splice site | absent | NP | absent |  |  | |
| **57** | 2-hom | c.2643+1G>A |  | splice site | absent | NP | NP |  |  | |
| **58** | 2-hom | c.4756C>T | p.Arg1586X | nonsense | NP | NP | NP |  |  | |
| **59** | 2-hom | c.1639-6G>A |  | splice site | NP | absent | NP |  |  | |
| **60** | 2-hom | c.5306C>A | p.Pro1769His | missense | reduced | NP | NP | not listed | 93 | |
| **61** | 2-hom | c.1392dupA | p.Asp465ArgfsX9 | frameshift | NP | NP | NP |  |  | |
| **62** | 2-hom | c.1020C>G | p.Ser340Arg | missense | NP | absent | NP | not listed | 71 | |
| **63** | 2-hom | c.5979dupA | p.Glu1994ArgfsX3 | frameshift | NP | NP | absent |  |  | |
| **64** | 2-hom | c.5979dupA | p.Glu1994ArgfsX3 | frameshift | NP | NP | NP |  |  | |
| **65** | 2-hom | c.4872_4876delGCCCGinsCCCC | p.Glu1624AspfsX9 | frameshift | NP | reduced | NP |  |  | |
| **66** | 2-hom | c.1663C>T | p. Arg555Trp | missense | NP | NP | absent | 0, 0.00004 | 88 | |
| **67** | 2-hom | c.2200_2205delACCCACinsT | Thr734SerfsX18 | frameshift | NP | absent | NP |  |  | |
| **68** | 2-hom | c.5200+1G>A |  | splice site | absent | absent | NP |  |  | |
| **69** | 2-hom | c.4253G>A | p.G1418D | missense | absent | NP | absent | 0,0 | 82 | |
| **70** | 2-hom | c.3444_3445 delinsAA | p.Tyr1148X | nonsense | NP | reduced | NP |  |  | |
| **71** | 2-hom | c.3444_3445 delinsAA | p.Tyr1148X | nonsense | NP | absent | NP |  |  | |
| **72** | 2-hom | c.2200_2204del | p.Thr734ProfsX18 | frameshift | NP | absent | NP |  |  | |
| **73** | 2-hom | c.3321-3322delinsC | p.Ala1108LeuFsx12 | frameshift | NP | NP | NP |  |  | |
| **74** | 2-hom | c.3321-3322delinsC | p.Ala1108LeuFsX12 | frameshift | absent | absent | NP |  |  | |
| **75** | 2-hom | c.2875C>T | p.Arg959Trp | missense | NP | NP | NP | 0, 0.0001 | 94 | |
| **76** | 2-hom | c.1861G>A | p.Gly621Arg | missense | reduced | normal | NP | not listed | 100 | |
| **77** | 2-hom | c.1368C>A | p.Cys456X | nonsense | absent | NP | absent |  |  | |
| **78** | 2-hom | c.457+1dupG | not available | splice site | NP | NP | NP |  |  | |
| **79** | 2-hom | c.2493_2496delACAG | p.Leu831LeufsX4 | frameshift | NP | NP | NP |  |  | |
| **80** | 2-hom | c.3992G>T | p.arg1331Leu | missense | reduced | absent | NP | 27, 0.02 | 82 | |
| **81** | 2-hom | c.1663C>T | p.R555W | missense | NP | NP | NP | 0, 0.00004 | 88 | |
| **82** | 2-hom | c.3121C>T | p.Arg1041Cys | missense | NP | absent | NP | 0,0.0002 | 94 | |
| **83** | 2-hom | c.1096DelA | p.Ser366AlafsX9 | frameshift | NP | NP | NP |  |  | |
| **84** | 2-het | c.827delA | p.Glu276GlyfsX12 | frameshift | reduced | absent | NP |  |  | |
|  |  | c.2643+1G>A |  | splice site |  |  |  |  |  | |
| 85 | 2-het | c.855+1delG |  | splice site | reduced | absent | NP |  |  | |
|  |  | c.3112C>T | p.Arg1038X | nonsense |  |  |  |  |  | |
| **86** | 2-het | c.107_108delAA | p.Lys36Serfsx12 | frameshift | reduced | absent | NP |  |  | |
|  |  | c.3028C>T | p.Gln1010X | nonsense |  |  |  |  |  | |
| **87** | 2-het | c.107_108delAA | p.Lys36Serfsx12 | frameshift | reduced | absent | NP |  |  | |
|  |  | c.3028C>T | p.Gln1010x | nonsense |  |  |  |  |  | |
| **88** | 2-het | c.5058-2A>G |  | splice site | NP | NP | NP |  |  | |
|  |  | c.856-3C>G |  | splice site |  |  |  |  |  | |
| **89** | 2-het | c.937+1G>C |  | splice site | NP | absent | NP |  |  | |
|  |  | c.3512_3513delCT | p.Ser1171PhefsX3 | frameshift |  |  |  |  |  | |
| **90** | 2-het | c.857T>A | p.Val286Glu | missense | NP | NP | NP | not listed | 99 | |
|  |  | c.3832C>T | p.Gln1278X | nonsense |  |  |  |  |  | |
| **91** | 2-het | c.3444_3445delinsAA | p.Tyr1148X | nonsense | reduced | NP | NP |  |  | |
|  |  | c.6124C>T | p.Arg2042Cys | missense |  |  |  | 0, 0.00004 | 93 | |
| **92** | 2-het | c.827delA | p.Glu276GlyfsX12 | frameshift | reduced | reduced | NP |  |  | |
|  |  | c.1861G>A | p.Gly621Arg | missense |  |  |  | not listed | 100 | |
| **93** | 2-het | c.3832C>T | p.Gln1278X | nonsense | absent | reduced | NP |  |  | |
|  |  | c.5979dupA | p.Glu1994ArgfsX3 | frameshift |  |  |  |  |  | |
| **94** | 2-het | c.3516_3517del | p.Ser1173X | nonsense | reduced | absent | NP |  |  | |
|  |  | c.4908_4936dup | p.Thr1646IlefsX8 | frameshift |  |  |  |  |  | |
| **95** | 2-het | c.5908C>T | p.Prol1970Ser | missense | absent | reduced | NP | 0,0 | 76 | |
|  |  | c.353delIT | p.Val118AlafsX33 | frameshift |  |  |  |  |  | |
| **96** | 2-het | c.3517dupT | p.Ser1173Phefsx2 | frameshift | absent | NP | NP |  |  | |
|  |  | c.5836-5839delCAGC | p.Gln1946Trpfsx19 | frameshift |  |  |  |  |  | |
| **97** | 2-het | c.855+1delG |  | splice site | absent | absent | NP |  |  | |
|  |  | c.3031G>C | p.Gly1011Arg | missense |  |  |  | 0,0 | 100 | |
| **98** | 2-het | c.3112C>T | p. Arg1038X | nonsense | NP | NP | absent |  |  | |
|  |  | c.3191_3196dup | p. Ala1064_Glu1065dup | inframe duplication |  |  |  |  |  | |
| **99** | 2-het | c.5979dup | p.Glu1994ArgfsX3 | frameshift | NP | NP | absent |  |  | |
|  |  | c.6124C>T | p.Arg2042Cys | missense |  |  |  | 0, 0.00004 | 93 | |
| **100** | 2-het | c.5979dup | p.Glu1994ArgfsX3 | frameshift | absent | absent | absent |  |  | |
|  |  | c.6124C>T | p.Arg2042Cys | missense |  |  |  | 0, 0.00004 | 93 | |
| **101** | 2-het | c.154T>C | p. Trp52Arg | missense | absent | absent | absent | 0,0 | 93 | |
|  |  | c.701G>A | p. Gly234Glu | missense |  |  |  | 0, 0.00004 | 86 | |
| **102** | 2-het | c. 5159delG | p.Arg1720LeufsX2 | frameshift | absent | absent | absent |  |  | |
|  |  | c.5979dupA | p.Glu1994ArgfsX3 | frameshift |  |  |  |  |  | |
| **103** | 2-het | c.3373delG | p.Glu1125LysfsX9 | frameshift | absent | NP | NP |  |  | |
|  |  | c.4748_4750delACAinsT | p.Tyr1583PhefsX17 | frameshift |  |  |  |  |  | |
| **104** | 2-het | c.2997G>T | p.Trp999Cys | missense | absent | NP | NP | 0, 0.00002 | 88 | |
|  |  | c.4497delT | p.Phe1499LeufsX4 | frameshift |  |  |  |  |  | |
| **105** | 2-het | c.2746_2747dupGG | p.Asp916GlyfsX33 | frameshift | absent | NP | NP |  |  | |
|  |  | c.5309T>C | p.Leu1770Pro | missense |  |  |  | 0,0 | 76 | |
| **106** | 2-het | c.5159delG | p.Arg1720LeufsX2 | frameshift | NP | NP | absent |  |  | |
|  |  | c.5979dupA | p.Glu1994ArgfsX3 | frameshift |  |  |  |  |  | |
| **107** | 2-het | c.3116G>T | p.Arg1039Leu | missense | absent | NP | NP | 0,0 | 88 | |
|  |  | c.5302C>T | p.Arg1768Trp | missense |  |  |  | 0, 0.000008283 | 94 | |
| **108** | 2-het | c.3116G-T | p.Arg1039Leu | missense | absent | NP | NP | 0,0 | 88 | |
|  |  | c.5302C-T | p.Arg1768Trp | missense |  |  |  | 0, 0.000008283 | 94 | |
| **109** | 2-het | c.3118C>T | p.Arg1040Trp | missense | absent | reduced | NP | 0,0 | 88 | |
|  |  | c.5979dupA | p.Glu1994ArgfsX3 | frameshift |  |  |  |  |  | |
| **110** | 2-het | c.4756C>T | p.Arg1586X | nonsense | absent | absent | NP |  |  | |
|  |  | c.5022delT | p.Phe1674LeufsX48 | frameshift |  |  |  |  |  | |
| **111** | 2-het | c.2367C>A | p.Ser789Arg | missense | absent | absent | NP | 0,0 | 82 | |
|  |  | c.5979dupA | p.Glu1994ArgfsX3 | frameshift |  |  |  |  |  | |
| **112** | 2-het | c.4756C>T | p.Arg1586X | nonsense | NP | NP | NP |  |  | |
|  |  | c.5022delT | p.Phe1674LeufsX48 | frameshift |  |  |  |  |  | |
| **113** | 2-het | c.757C>T | p.Arg253Trp | missense | absent | NP | NP | 0, 0.0001 | 88 | |
|  |  | c.5979dupA | p.Glu1994ArgfsX3 | frameshift |  |  |  |  |  | |
| **114** | 2-het | c.855+1del G |  | splice site | absent | absent | NP |  |  | |
|  |  | c.895G>A | p.Gly299Arg | missense |  |  |  | not listed | 100 | |
| **115** | 2-het | c.1566C>G | p.Tyr522X | nonsense | absent | NP | NP |  |  | |
|  |  | c.2997G>T | p.Trp999Cys | missense |  |  |  | 0, 0.00002 | 88 | |
| **116** | 2-het | c.1566C>G | p.Tyr522X | nonsense | absent | absent | NP |  |  | |
|  |  | C.5698_5699delAG | p.Ser1900GlnfsX14 | frameshift |  |  |  |  |  | |
| **117** | 2-het | c.2997G>T | p.Trp999Cys | missense | absent | absent | NP | 0, 0.00002 | 88 | |
|  |  | c.6135G>A | p.Trp2045X | nonsense |  |  |  |  |  | |
| **118** | 2-het | c.2997G>T | p.Trp999Cys | missense | absent | absent | NP | 0, 0.00002 | 88 | |
|  |  | c.6135G>A | p.Trp2045X | nonsense |  |  |  |  |  | |
| **119** | 2-het | c.6135G>A | p.Trp2045X | nonsense | absent | absent | NP |  |  | |
|  |  | c.342+1G>A |  | splice site |  |  |  |  |  | |
| **120** | 2-het | c.796_797delCT | p.Leu266PhefsX6 | frameshift | NP | NP | NP |  |  | |
|  |  | c.2777G>A | p.W926X | nonsense |  |  |  |  |  | |
| **121** | 2-het | c.796_797delCT | p.Leu266PhefsX6 | frameshift | absent | absent | NP |  |  | |
|  |  | c.2777G>A | p.Trp926x | nonsense |  |  |  |  |  | |
| **122** | 2-het | c.5979dupA | p.Glu1994ArgfsX3 | frameshift | NP | NP | NP |  |  | |
|  |  | c.487c>T | p.Gln163X | nonsense |  |  |  |  |  | |
| **123** | 2-het | c.353delIT | p.Val118AlafsX33 | frameshift | absent | absent | NP |  |  | |
|  |  | c.1343T>C | p.Leu448Pro | missense |  |  |  | 0,0 | 69 | |
| **124** | 2-het | c.3517dupT | p.Ser1173PhefxX2 | frameshift | NP | NP | NP |  |  | |
|  |  | c.4872_4876delinsCCCC | p.Glu1624AspfsX10 | frameshift |  |  |  |  |  | |
| **125** | 2-het | c.865T>C | p.Ser289Pro | missense | NP | NP | normal | 0,0 | 80 | |
|  |  | c.4794G>T | p.Lys1598Asn | missense |  |  |  | 0,0 | 100 | |
| **126** | 2-het | c.1392dupA | p.Asp465ArgfsX9 | frameshift | NP | NP | absent |  |  | |
|  |  | c.5713C>T | p.Arg1905X | nonsense |  |  |  |  |  | |
| **127** | 2-het | c.6124C>T | p.Arg2042Cys | missense | absent | NP | NP | 0, 0.00004 | 93 | |
|  |  | c.4408C>T | p.Gln1470X | nonsense |  |  |  |  |  | |
| **128** | 2-het | c.1638+2T>A |  | splice site | NP | NP | <20% |  |  | |
|  |  | c.1642delG | p.Glu548LysfxX79 | frameshift |  |  |  |  |  | |
| **129** | 2-het | c.5429+1G>T |  | splice site | NP | NP | NP |  |  | |
|  |  | c.5057+5G>T |  | splice site |  |  |  |  |  | |
| **130** | 2-het | c.855+1delG | splice donar deletion | splice site | absent | NP |  |  |  | |
|  |  | c.3036G>A | p.Trp1012X | nonsense |  |  |  |  |  | |
| **131** | 2-het | c.3444_3445delTGinsAA | p.Tyr1148X | nonsense | reduced | NP | NP |  |  | |
|  |  | c.2512-1495_3148del | p.Tyr838SerfsX7 | frameshift |  |  |  |  |  | |
| **132** | 2-het | c.1398-1G>A |  | splice site | NP | absent | NP |  |  | |
|  |  | c.2071C>T | p.Gln691X | nonsense |  |  |  |  |  | |
| **133** | 2-het | c.1392dupA | p.Asp465ArgfsX9 | frameshift | absent | absent | NP |  |  | |
|  |  | c.3516_3517delTT | p.Ser1173X | nonsense |  |  |  |  |  | |
| **134** | 2-het | c.755c>t | p.Thr252Met | missense | absent | reduced | NP | 0,0 | 88 | |
|  |  | c.1464delIT | p.Gly489GlufsX4 | frameshift |  |  |  |  |  | |
| **135** | 2-het | c.855+1delG |  | splice site | NP | NP |  |  |  | |
|  |  | c.3505insC | p.Lys1169GlnfsX6 | frameshift |  |  |  |  |  | |
| **136** | 2-het | c.1020C>G | p.Ser340Arg | missense | reduced | reduced | NP | not listed | 71 | |
|  |  | c.4883G>T | p.Gly1628Val | missense |  |  |  | 0,0 | 82 | |
| **137** | 2-het | c.1932dupG | p.Cys645ValfsX10 | frameshift | absent | NP | NP |  |  | |
|  |  | c.3832C>T | p.Gln1278X | nonsense |  |  |  |  |  | |
| **138** | 2-het | c.5911T>C | p.Cys1971Arg | missense | NP | NP | NP | not listed | 92 | |
|  |  | c.5713C>T | p.Arg1905X | nonsense |  |  |  |  |  | |
| **139** | 2-het | c.4894G>T | p.Glu1632X | nonsense | reduced | reduced | 1% |  |  | |
|  |  | c.1398-2A>G | splicing | splice site |  |  |  |  |  | |
| **140** | 2-het | c.526C>T | p.Gln176X | nonsense | absent | absent | NP |  |  | |
|  |  | c.2858dupT | p.Phe954ValfsX2 | frameshift |  |  |  |  |  | |
| **141** | 2-het | c.2372dupC | p.Asp792GlyfsX73 | frameshift | reduced | reduced | absent |  |  | |
|  |  | c.4090C>T | p.Gln1364X | nonsense |  |  |  |  |  | |
| **142** | 2-het | c.610C>T | p.Arg204X | nonsense | NP | NP | absent |  |  | |
|  |  | c.4194insC | p.Ile1401HisfsX8 | frameshift |  |  |  |  |  | |
| **143** | 2-het | c.879_883dupGACAG | p.Asp295GlyfsX45 | frameshift | reduced | NP | 1% |  |  | |
|  |  | c.5768-?_5946+?del | p.Ser1924GlnfsX13 | frameshift |  |  |  |  |  | |
| **144** | 2-het | c.896G>A | p.Gly299Glu | missense | reduced | reduced | NP | 0,0 | 99 | |
|  |  | c.1877T>C | p.Met 626Thr | missense |  |  |  | 0, 0.0015 | 71 | |
| **145** | 2-het | c.2643+1G>A |  | splice site | NP | NP | absent |  |  | |
|  |  | c.3327_3328delGT | p.Phe1110CysfsX3 | frameshift |  |  |  |  |  | |
| **146** | 2-het | c.1481-1G>A |  | splice site | NP | NP | absent |  |  | |
|  |  | c.5713C>T | p.Arg1905X | nonsense |  |  |  |  |  | |
| **147** | 2-het | c.757C>T | Arg253Trp | missense | absent | absent | NP | 0, 0.0001 | 93 | |
|  |  | c.4824delG | p.Lys1609ArgfsX4 | frameshift |  |  |  |  |  | |
| **148** | 2-het | c.3805dupG | p.Glu1269GlyfsX7 | frameshift | absent | absent | NP |  |  | |
|  |  | c.5698_5699delAG | p.Ser1900GlnfsX14 | frameshift |  |  |  |  |  | |
| **149** | 2-het | c.5668-7G>A |  | splice site | reduced | absent | 10% |  |  | |
|  |  | c.5768_5946del | p.Ser1924GlnfsX13 | frameshift |  |  |  |  |  | |
| **150** | 2-het | c.797T>G | p.Leu266Arg | missense | NP | NP | absent | 0,0 | 86 | |
|  |  | c.1663C>T | p.Arg555Trp | missense |  |  |  | 0, 0.00004 | 88 | |
| **151** | 2-het | c.3444_3445 delines AA | p.Tyr1148x | nonsense | NP | NP | NP |  |  | |
|  |  | c.5903 G>A | p.Trp1968x | nonsense |  |  |  |  |  | |
| **152** | 2-het | c.509C>A | p.Ala170Glu | missense | absent | normal | NP | 8, 0.0098 | 71 | |
|  |  | c.5836_5839delCAGC | p.Gln1946TrpfsX19 | frameshift |  |  |  |  |  | |
| **153** | 2-het | c.490G>T | p.Gly164X | nonsense | absent | absent | NP |  |  | |
|  |  | c.3703-1G>A |  | splice site |  |  |  |  |  | |
| **154** | 2-het | c.855+1delG | altered donon of intron 8 | splice site | absent | absent | NP |  |  | |
|  |  | c.5594delG | p.Gly1865Alafs*101 | frameshift |  |  |  |  |  | |
| **155** | 2-het | c.509C>A | p.Ala170Glu | missense | absent | absent | NP | 8, 0.0098 | 71 | |
|  |  | c.1663C>T | p.Arg555Trp | missense |  |  |  | 0, 0.00004 | 88 | |
| **156** | 2-het | c.1392dupA | p.Asp465ArgfsX9 | frameshift | NP | NP | NP |  |  | |
|  |  | c.1481-1G>A | splicing | splice site |  |  |  |  |  | |
| **157** | 2-het | c.3113G>C | p.Arg1038Pro | missense | absent | NP | absent | 0,0 | 88 | |
|  |  | c.4756C>T | p.Arg1586X | nonsense |  |  |  |  |  | |
| **158** | 2-het | c.664-2A.G |  | splice site | NP | NP | absent |  |  | |
|  |  | C.2311C>T | p.Gln771X | nonsense |  |  |  |  |  | |
| **159** | 2-het | c.757C>T | Arg253Trp | missense | NP | NP | NP | 0, 0.0001 | 93 | |
|  |  | c.4824delG | p.Lys1609ArgfsX4 | frameshift |  |  |  |  |  | |
| **160** | 2-het | c.3992G>T | p.Arg1331Leu | missense | NP | NP | normal | 27, 0.02 | 82 | |
|  |  | c.3065G>A | p.Arg1022Gln | missense |  |  |  | 29, 0.02 | 65 | |
| **161** | 2-het | c.1392dupA | p.Asp465Argfs*9 | frameshift | NP | NP | NP |  |  | |
|  |  | c.5698_5699delAG | p.Ser1900GlnfsX14 | frameshift |  |  |  |  |  | |
| **162** | 2-het | c.1168G>T | p.Asp390Tyr | missense | absent | absent | NP | 0,0 | 95 | |
|  |  | c.1177C>T | p.Gln393 X | nonsense |  |  |  |  |  | |
| **163** | 2-het | c.2352_2355+1delGGAGG |  | splice site | reduced | NP | absent |  |  | |
|  |  | c.6124C>T | p.R2042C | missense |  |  |  | 0,0.00004 | 93 | |
| **164** | 2-het | c.610C>T | p.Arg204X | nonsense | absent | NP | absent |  |  | |
|  |  | c.1053+1G>A |  | splice site |  |  |  |  |  | |
| **165** | 2-het | c.2772G>A | p.trp924X | nonsense |  |  |  |  |  | |
|  |  | c.4872_4876delinsCCCC | p.Glu1624AspfsX10 | frameshift |  |  |  |  |  | |
| **166** | 2-het | c.610C>T | p.R204X | nonsense | NP | reduced | NP |  |  | |
|  |  | c.5979dupA | p.Glu1994ArgfsX3 | frameshift |  |  |  |  |  | |
| **167** | 2-het | c.757C>T | p.Arg253Trp | missense | NP | NP | absent | 0, 0.0001 | 93 | |
|  |  | c.5871-5872delGT | p.Ser1958ProfsX3 | frameshift |  |  |  |  |  | |
| **168** | 2-het | C. 20T >C | p. Leu7 Pro | missense |  |  |  | 0,0 | 65 | |
|  |  | C. 2810C >T | p. Thr 937 Ile | missense |  |  |  | 1, 0.0001 | 79 | |
| **169** | 2-het | c.5077C>T | p.Arg1693Trp | missense |  |  |  | 0,0 | 88 | |
|  |  | c.5698_5699delAG | p.Ser1900GlnfsX14 | frameshift |  |  |  |  |  | |
| **170** | 2-het | c.2077delC | p.His693Thrfsx4 | frameshift | NP | absent | NP |  |  | |
|  |  | c.4334-3C>A |  | splice site |  |  |  |  |  | |
| **171** | 2-het | c.490G>T | p.Gly164X | nonsense | NP | NP | NP |  |  | |
|  |  | c.3703-1G>A |  | splice site |  |  |  |  |  | |
| **172** | 2-het | c.610C>T | p.Arg204X | nonsense | NP | NP | absent |  |  | |
|  |  | c.1053+1G>A |  | splice site |  |  |  |  |  | |
| **173** | 2-het | c.1595dupT | p. Thr 533 His fsX 37 | frameshift | NP | NP | NP |  |  | |
|  |  | c.3444_3445delTGinsAA | p. Tyr 1148 X | nonsense |  |  |  |  |  | |
| **174** | 2-het | c.2077delC | p.His693ThrfsX4 | frameshift | NP | reduced | absent |  |  | |
|  |  | c.3121C>T | p.Arg1041Cys | missense |  |  |  | 0,0.0002 | 94 | |
| **175** | 2-het | c.5644C>T | p.Gln1882X | nonsense | reduced | absent | NP |  |  | |
|  |  | c.5836_5839delCAGC | p.Gln1946TrpfsX19 | frameshift |  |  |  |  |  | |
| **176** | 2-het | c.2077delC | p.His693ThrfsX4 | frameshift | NP | reduced | NP |  |  | |
|  |  | c.2512_3174del | p.Tyr838_Arg1058del | inframe deletion |  |  |  |  |  | |
| **177** | 2-het | c.2200_2205delinsT | Thr734SerfsX18 | frameshift | NP | absent | NP |  |  | |
|  |  | c.3516_3517delTT | Ser1173X | nonsense |  |  |  |  |  | |
| **178** | 2-het | c.5497G>T | p.E1833X | nonsense | absent | absent | absent |  |  | |
|  |  | c.5946+1G>A |  | splice site |  |  |  |  |  | |
| **179** | 2-het | c.115_125dupAAbAACAGCGT | p.Asn43ArgfsSTOP4 | frameshift | NP | NP | absent |  |  | |
|  |  | c.2974T>C | p.Trp992Arg | missense |  |  |  | 0, 0.00001 | 88 | |
| **180** | 2-het | c.3112C>T | p.R1038X | nonsense | NP | NP | NP |  |  | |
|  |  | C.6008G>A | p.G2003D | missense |  |  |  | 0, 0.00004 | 88 | |
| **181** | 2-het | c.4299C>G | p.Y1433X | nonsense | absent | absent | NP |  |  | |
|  |  | c.5776C>T | p.R1926X | nonsense |  |  |  |  |  | |
| **182** | 2-het | c.525delT | Gln176Lysfs*51 | frameshift | NP | NP | NP |  |  | |
|  |  | c.5979dupA | Glu1994Argfs*3 | frameshift |  |  |  |  |  | |
| **183** | 2-het | c.525detT | Gln176Lysfs*51 | frameshift | NP | NP | NP |  |  | |
|  |  | c.5979dupA | Glu1994Argfs*3 | frameshift |  |  |  |  |  | |
| **184** | 2-het | c.525delT | Gln176Lysfs*51 | frameshift | NP | NP | NP |  |  | |
|  |  | c.5979dupA | Glu1994Argfs*3 | frameshift |  |  |  |  |  | |
| **185** | 2-het | c.2779delG | p.Ala927LeufsX21 | frameshift | NP | NP | NP |  |  | |
|  |  | c.4253G>A | p.Gly1418Asp | missense |  |  |  | 0,0 | 82 | |
| **186** | 2-het | c.3516_3517del; | p.Ser1173X | nonsense | absent | NP | NP |  |  | |
|  |  | c.4411-5C>G |  | splice site |  |  |  |  |  | |
| **187** | 3-het | c.1343T>C | p.Leu448Pro | missense | NP | absent | NP | 0,0 | 69 | |
|  |  | c.3444_3445delinsAA | p.Tyr1148X | nonsense |  |  |  |  |  | |
|  |  | c.3992G>T | p.Arg1331Leu | missense |  |  |  | 27, 0.02 | 82 | |
| **188** | 3-het | c.3444T>A | p.Tyr1148X | nonsense | reduced | absent | NP |  |  | |
|  |  | c.4756C>T | p.Arg1586X | nonsense |  |  |  |  |  | |
|  |  | c.3445G>A | p.Gly1149Arg | missense |  |  |  | 0, 0.00001 | 82 | |
| **189** | 3-het | c.2077delC | p.His693Thrfsx4 | frameshift | NP | absent | NP |  |  | |
|  |  | c.3121C>T | p.Arg1041Cys | missense |  |  |  | 0,0.0002 | 94 | |
|  |  | c.6056G>A | p.Arg2019Lys | missense |  |  |  | 0,0 | 71 | |
| **190** | 3-het | c.2643+1G>A |  | splice site | NP | NP | NP |  |  | |
|  |  | c.3112C>T | p.Arg1038X | nonsense |  |  |  |  |  | |
|  |  | c.4577A>C | p.Lys1526Thr | missense |  |  |  | 0,0.0002 | 80 | |
| **191** | 3-het | c.2643+1G>A |  | splice site | reduced | NP | absent |  |  | |
|  |  | c.4577A>C | p.Lys1526Thr | missense |  |  |  | 0,0.0002 | 80 | |
|  |  | c.2790G>C | p.Trp930Cys | missense |  |  |  | 0,0 | 82 | |
| **192** | 4-hom | c.3041A>G | p.Tyr1014Cys | missense | NP | NP | NP | 0,0.00003 | 82 | |
|  |  | c.3041A>G | p.Tyr1014Cys | missense |  |  |  | 0,0.00003 | 82 | |
|  |  | c.4820T>C | (p.Ile1607Thr | missense |  |  |  | 4, 0.003 | 94 | |
|  |  | c.4820T>C | (p.Ile1607Thr | missense |  |  |  | 4, 0.003 | 94 | |
| **193** | 4-hom | c.3041A>G | p.Tyr1014Cys | missense | NP | NP | NP | 0, 0.00003 | 82 | |
|  |  | c.3041A>G | p.Tyr1014Cys | missense |  |  |  | 0, 0.00003 | 82 | |
|  |  | c.4820T>C | c.IIe1607Thr | missense |  |  |  | 4,0.003 | 94 | |
|  |  | c.4820T>C | c.IIe1607Thr | missense |  |  |  | 4,0.003 | 94 | |
