# Supplementary material for: The Clinical Outcome Study for dysferlinopathy: An international multicenter study
Source: Neurol Genet. 2016 Aug 4;2(4):e89. doi: 10.1212/NXG.0000000000000089 (PMC4994875; doi:10.1212/NXG.0000000000000089)
Supplement: Data Supplement [file supp_2.4.e89_table_e-2.docx]

**Supplementary table 2 - Adapted North Star Ambulatory Assessment for Dysferlinopathy**

| *Activity\Score* | 4 | *3* | **2** | **1** | **0** |
| --- | --- | --- | --- | --- | --- |
| **1. Stand** |  |  | Stands upright and symmetrically, without compensation (with heels flat and legs in neutral) for minimum count of 3 seconds | Stands but with some degree of compensation | Cannot stand independently, needs support |
| **2. Walk (10 m)** |  |  | Walks consistently with heel-toe or flat-footed gait pattern | Adapted walking pattern  e.g. wide base, altered foot posture, other please define in comments. | Loss of independent ambulation – may use knee‑ankle‑foot orthosis (KAFO) or walk with assistance |
| **3. Stand up from chair** |  |  | Keeping arms folded.  Starting position 90˚ hips and knees, feet on floor/ supported on a box step | With help from thighs or push on chair or prone turn or alters position by widening base | Unable |
| **4. Stand on one leg - right** |  |  | Able to stand in a relaxed manner (no fixation) for count of 3 seconds | Stands but either momentarily or needs a lot of fixation e.g. by knees tightly adducted or other trick | Unable |
| **5. Stand on one leg - left** |  |  | Able to stand in a relaxed manner (no fixation) for count of 3 seconds | Stands but either momentarily or needs a lot of fixation e.g. by knees tightly adducted or other trick | Unable |
| **6. Climb box step - right** |  |  | Faces step – no support needed | Goes up sideways or needs support | Unable |
| **7. Climb box step - left** |  |  | Faces step – no support needed | Goes up sideways or needs support | Unable |
| **8. Descend box step -right** |  |  | Faces forward, climbs down controlling weight bearing leg. No support needed | Sideways, skips down or needs support | Unable |
| **9. Descend box step -left** |  |  | Faces forward, climbs down controlling weight bearing leg. No support needed | Sideways, skips down or needs support | Unable |
| 10. Lifts head |  |  | In supine, head must be lifted in mid-line. Chin moves towards chest | Head is lifted but through side flexion or with no neck flexion | Unable |
| **11. Gets to sitting** |  |  | Starts in supine – may use one hand to assist | Self assistance, e.g.: pulls on legs or uses head-on-hands or head flexed to floor | Unable |
| **12. Rise from floor** |  |  | Starts in long sitting - No evidence of Gower’s manoeuvre. | Exhibits at least one of the components described above – in particular rolls towards floor, and/**or** use hand(s) on legs | (a) NEEDS to use external support object e.g. chair OR (b) Unable |
| **13. Stands on heels** |  |  | Both feet at the same time, clearly standing on heels only (acceptable to move a few steps to keep balance) for count of 3 seconds | Flexes hip and only raises forefoot | Unable |
| 14. Jump |  |  | Both feet at the same time, clears the ground simultaneously | One foot after the other (skip) | Unable |
| **15. Hop right leg** |  |  | Clears forefoot and heel off the floor | Able to bend knee, no floor clearance | Unable |
| **16. Hop left leg** |  |  | Clears forefoot and heel off the floor | Able to bend knee, no floor clearance | Unable |
| **17. Run (10 m)** |  |  | Can run the distance - both feet off the ground - no double stance phase during running | Speeds up walk but maintains double stance phase | Walks with no extra speed  OR  Unable to  walk 10 m |
| 18. Squat down | Squats down fully with arms free (more than 90° of hip and knee flexion) | Uses one or two hands on thighs or floor to assist full squat | Able to squat half way more than 20˚ but less than 90˚ hip and knee flexion with or without hands | Able to initiate hip and knee flexion to no more than 20˚ | Unable |
| 19. Rise from squat |  | Stands up from full squat without using arms / hands on floor or legs | Uses one or two hands on thighs to get up from full squat to stand | Uses one or two hands on floor to get up from full squat | Unable to get back up from squat without assistance  Or  Changes position (onto knees) to rise from squat |
| 20. High kneel to stand through R |  | Able with arms free | Able to stand up through R half kneeling using hands on floor or legs | Able to shift weight off both knees (with or without arm support)  But unable to fully rise even with arm support | Unable |
| 21. High kneel to stand through L |  | Able with arms free | Able to stand up through L half kneeling using hands on floor or legs | Able to shift weight off both knees (with or without arm support) but unable to fully rise even with arm support | Unable |
| 22. Tiptoes |  | Both feet at the same time, clearly on toes for count of 3 | Up on toes but only momentarily or just lifting heels | Up on forefoot only (heel raised) with or without knee flexion | Unable |

**Supplementary table 2:** Total score is calculated by adding up all the components of this assessment.
