# Supplementary material for: The Clinical Outcome Study for dysferlinopathy: An international multicenter study
Source: Neurol Genet. 2016 Aug 4;2(4):e89. doi: 10.1212/NXG.0000000000000089 (PMC4994875; doi:10.1212/NXG.0000000000000089)
Supplement: Coinvestigators [file supp_2.4.e89_coinvestigators.docx]

**Coinvestigators- The Jain COS Consortium**

Adrienne Arrieta MS (Children's National Medical Center (Washington DC, Data Management and Training)

Jia Feng MPH (Children's National Medical Center, Washington DC, Data Management)

Esther Hwang (Jain Foundation, Seattle, USA- Recruitment, development of assessment forms)

Elaine Lee PhD (Jain Foundation, Seattle, USA- Recruitment, development of assessment forms)

Isabel Illa MD (Hospital de la Santa Creu i Sant Pau/ CIBERER, Barcelona, Spain- Site Investigator)

Eduard Gallardo MD (Hospital de la Santa Creu i Sant Pau/ CIBERER, Barcelona, Spain- Site Investigator)

Irene Pedrosa Hernández PT (Servei de Medicina Física i Rehabilitació, Hospital de la Santa Creu i Sant Pau- Site Investigator)

Izaskun Belmonte Jimeno PT (Servei de Medicina Física i Rehabilitació, Hospital de la Santa Creu i Sant Pau Site Investigator)

Elke Maron (Elan-Physio, Berlin, Germany, Site Investigator)

Juliana Prügel (Elan-Physio, Berlin, Germany, Site Investigator)

Mohammed Sanjak PhD, PT, MBA (Carolinas HealthCare System, Charlotte, NC, Site Investigator)

Scott Holsten PT (Carolinas HealthCare System, Charlotte, NC, Site Investigator)

Jackie Sykes RN, BSN (Carolinas HealthCare System, Charlotte, NC, Study Coordinator)

Linda P Lowes, Ph.D (Nationwide Children’s Hospital, Columbus, OH, Site Investigator)

Lindsay Alfano, PT (Nationwide Children’s Hospital, Columbus, OH, Site Investigator)

Katherine Berry, PT (Nationwide Children’s Hospital, Columbus, OH, Site Investigator)

Brent Yetter, MS(Nationwide Children’s Hospital, Columbus, OH, Study Coordinator

Bendahan David  Phd (Centre de Résonance Magnétique Biologique et Médicale, UMR CNRS 7339, Aix-Marseille Université, Marseille, Site Investigator)

Bernard Lapeyssonie  PT (Neuromuscular and ALS center, La Timone Hospital, Aix-Marseille Université, Marseille, Site Investigator)

Attarian Shahram, MD, PhD (Neuromuscular and ALS center, La Timone Hospital, Aix-Marseille Université, Marseille, Site Investigator)

Testot-Ferry Albane, CRA (Neuromuscular and ALS center, La Timone Hospital, Aix-Marseille Université, Marseille, Study Coordinator)

Sabine Krause MD, (Friedrich-Baur-Institute, Munich, Site Investigator)

Simone Thiele PT (Friedrich-Baur-Institute, Munich, Site Investigator)

Robert Muni Lofra (The John Walton Muscular Dystrophy Research Centre, Newcastle upon Tyne, Site Investigator)

Luca Bello, (University of Padova, Site investigator)

Chiara Calore, (University of Padova, Site investigator)

Teresa Gidaro (Institut de Myologie, Paris, Site Investigator)

Laurent Servais (Institut de Myologie, Paris, Site Investigator)

Aurélie Canal PT (Institut de Myologie, Paris, Site Investigator)

Gwenn Ollivier PT (Institut de Myologie, Paris, Site Investigator)

Valérie Decostre PT (Institut de Myologie, Paris, Site Investigator)

Jean-Yves Hogrel (Institut de Myologie, Paris, Site Investigator)

Oumar Diabaté (Institut de Myologie, Paris, Study Coordinator)

Julaine Florence PT Phd(Washington University, St. Louis, MO, USA Project Manager)

Linda Schimmoeller (Washington University, St. Louis, MO, USA, Study Coordinator)

Catherine Siener PT (Washington University, St. Louis, MO, USA, Site Investigator)

Jeanine Schierbecker PT (Washington University, St. Louis, MO, Site Investigator )

Bosco Méndez MD (Hospital U. Virgen del Rocío/Instituto de Biomedicina de Sevilla Seville, co-IP)

Pilar Carbonell MD (Hospital U. Virgen del Rocío/Instituto de Biomedicina de Sevilla Seville, Site Investigator)

Nieves Sanchez-Aguilar PT (Hospital U. Virgen del Rocío/Instituto de Biomedicina de Sevilla Seville, Site Investigator)

Susana Rico MD (Hospital U. Virgen de Valme, Site Investigator)

Macarena Cabrera MD (Hospital U. Virgen del Rocío/Instituto de Biomedicina de Sevilla, Seville, Site Investigator)

| Yolanda Morgado MD (Hospital U. Virgen de Valme /Instituto de Biomedicina de Sevilla, Seville, Site Investigator) |
| --- |

Richard Gee, PT, (Stanford University Children’s Health, Stanford, Site Investigator)

Tina Duong, PT (Stanford University School of Medicine, Stanford, Site Investigator/ Trainer)

Jennifer Perez(Stanford University School of Medicine, Stanford, Study Coordinator)

Nigel F Clarke MD (Institute for Neuroscience and Muscle Research, Sydney Site Investigator)

Sarah Sandaradura MD (Institute for Neuroscience and Muscle Research, Sydney Site Investigator)

Roula Ghaoui MD (Institute for Neuroscience and Muscle Research, Sydney Site Investigator)

Kayla Cornett Ex Phys (Institute for Neuroscience and Muscle Research, Sydney Site Investigator)

Clare Miller PT(Institute for Neuroscience and Muscle Research, Sydney Site Investigator)

Meghan Harman PT(Institute for Neuroscience and Muscle Research, Sydney Site Investigator)

Kristy Rose PT Phd (Institute for Neuroscience and Muscle Research, Sydney Site Investigator/ Trainer)

Noriko Sato MD PhD (National Center of Neurology and Psychiatry, Tokyo, Site Investigator)

Takeshi Tamaru (National Center of Neurology and Psychiatry, Tokyo, Site Investigator)

Yoko Kobayashi MD(National Center of Neurology and Psychiatry, Tokyo, Site Investigator)

Hiroyuki Yajima PT (National Center of Neurology and Psychiatry, Tokyo, Site Investigator)

Chikako Sakamoto PT (National Center of Neurology and Psychiatry, Tokyo, Site Investigator)

Takayuki Tateishi PT (National Center of Neurology and Psychiatry, Tokyo, Site Investigator)

Ai Ashida PT (National Center of Neurology and Psychiatry, Tokyo, Site Investigator)

Takahiro Nakayama MD PhD (Yokohama Rosai Hospital, Yokohama, Study Advisor)

Kazuhiko Segawa MD PhD (National Center of Neurology and Psychiatry, Tokyo, Site Investigator)

Sachiko Ohtaguro (National Center of Neurology and Psychiatry, Tokyo, Study Assistant)

Harumasa Nakamura MD(National Center of Neurology and Psychiatry, Tokyo, Study Advisor)

Maki Ohhata (National Center of Neurology and Psychiatry, Tokyo, Study Coordinator)

En Kimura MD PhD(National Center of Neurology and Psychiatry, Tokyo, Study Advisor)

Makiko Endo (National Center of Neurology and Psychiatry, Tokyo, Study Coordinator)

Brittney Drogo PT, DPT (Children's National Health System, Washington, Site Investigator)

Nora Brody, PT, DPT(Children's National Health System, Washington, Site Investigator)

Meganne E Leach, MSN, APRN (Children's National Health System, Washington, Site Investigator)

Allyn Toles (Children's National Health System, Washington, Study Coordinator)

Heather Hilsden (The John Walton Muscular Dystrophy Research Centre, Newcastle upon Tyne, Project Manager)
